# Supplementary material for: Coherence protection of spin qubits in hexagonal boron nitride
Source: Nat Commun. 2023 Jan 28;14:461. doi: 10.1038/s41467-023-36196-7 (PMC9884286; doi:10.1038/s41467-023-36196-7)
Supplement: Supplementary file 1 — Supplementary Information [file 41467_2023_36196_MOESM1_ESM.pdf]

Supplementary Information: Coherence protection of spin qubits in hexagonal boron nitride

Andrew J. Ramsay[1], Reza Hekmati[2], Charlie J. Patrickson[3], Simon Baber[3], David R. M. Arvidsson-Shukur[1], Anthony J Bennett[2,4], and Isaac J. Luxmoore[3,*]

[1]*Hitachi Cambridge Laboratory, Hitachi Europe Ltd., CB3 0HE,UK*
[2]*School of Physics and Astronomy, Cardiff University, Queen's Building, CF24 3AA, UK*
[3]*Department of Engineering, University of Exeter, EX4 4QF, UK*
[4]*School of Engineering, Cardiff University, Queen's Building, CF24 3AA, UK*
[*]*corresponding author: Isaac J. Luxmoore (i.j.luxmoore@exeter.ac.uk)*

Supplementary Note 1: Theory of concatenated continuous dynamic decoupling

The Hamiltonian of the $S = 1$ crystal ground-state can be expressed as H_e.

$$H_e = DS_z^2 + E(S_x^2 - S_y^2) + \gamma_e B_z S_z, \tag{1}$$

is composed of a zero-field splitting with $D = 3.47$ GHz, and $E \approx 59$ MHz, see Supplementary Fig. 2, and an electron Zeeman term with gyromagnetic ratio $\gamma_e \approx 28$ MHz/mT. S_j are the S=1 electron spin operators. The D-term defines a quantisation-axis along the c-axis of the crystal, and the static B-field is applied along this direction. To control the spin, an alternating magnetic-field is applied along the x-axis, and has the form $H_c(t)$.

$$H_c(t) = [\Omega \cos(\omega t + \phi) + 2\epsilon_m \sin(\omega t + \phi) \sin(\omega_m t - \theta_m)]S_x \tag{2}$$

The first term is the usual Rabi drive. The second term adds an amplitude modulated field in quadrature with the Rabi drive. To simplify the discussion we consider only a two-level system comprised of the $m_s = 0, m_s = +1$ states that are near resonance with the Rabi drive, i.e. $S \rightarrow \sigma$, $H_e \rightarrow \frac{1}{2}\omega_0\sigma_z$.

Transforming to the first rotating frame of the Rabi drive: $H_c^{'} = e^{i\omega t\sigma_z/2} H_c e^{-i\omega t\sigma_z/2}$. In the case $\phi = 0$,

$$H_c^{'} = \frac{1}{2}[\Omega\sigma_x^{'} - 2\epsilon_m \sin(\omega_m t - \theta_m)\sigma_y^{'} + (\omega_0 - \omega)\sigma_z^{'}] \equiv \frac{1}{2}\mathbf{\Omega_{eff}^{'}(t)}.\sigma^{'} \tag{3}$$

where $\sigma_\alpha^{'}$ are the Pauli spin-1/2 matrices, and the superscript identifies the frame. The counter-rotating term has been neglected. The Hamiltonian can be expressed as an effective magnetic field $\Omega_{eff}^{'}(t)$ that causes the spin-1/2 to rotate. We consider the resonant case, where the detuning $\Delta = \omega_0 - \omega = 0$. If $\epsilon_m = 0$, the Ω-term drives an unprotected Rabi oscillation about the $x^{'}$-axis. In this frame, the ϵ_m term provides an AC B-field, that causes the direction of the effective magnetic field to rock in the $x^{'}y^{'}$-plane, as will be discussed later.

Making a second rotating frame approximation to rotate into the dressed states basis, $H_c^{''} = e^{i\omega_m t\sigma_x^{'}/2} H_c^{'} e^{-i\omega_m t\sigma_x^{'}/2} - \omega_m\sigma_x^{'}/2$.

$$H_c^{''} = \frac{1}{2}(\Omega - \omega_m) + \frac{\epsilon_m}{2}[\sin(\theta_m)[-\sin(\phi)\sigma_x^{''} + \cos(\phi)\sigma_y^{''}] + \cos(\theta_m)\sigma_z^{''}] \equiv \frac{1}{2}\mathbf{\Omega_{eff}^{''}(t)}.\sigma^{''} \tag{4}$$

In the second rotating frame, the control field is equivalent to a DC magnetic field $\Omega^{''}_{eff}(t)$ of magnitude ϵ_m that is oriented using the phases of the drive θ_m and ϕ. By setting the drive such that $\Omega = \omega_m$, $\theta_m = \phi = 0$, the Hamiltonian reduces to $H^{''}_C = \frac{1}{2}\epsilon_m\sigma^{''}_z$, and we can define a protected basis. In the following sections, we will present a qualitative discussion of how this control field can protect against decoherence.

Supplementary Note 2: Locking of Rabi frequency to modulation frequency, ω_m

We start by considering the damping of an unprotected single-drive Rabi oscillation ($\epsilon_m = 0$) due to low frequency fluctuations in the Rabi frequency, most likely caused by errors in the microwave power applied. To this end we numerically calculate the Bloch-vector dynamics in the first rotating frame with no damping using the Heisenberg equation $\dot{\sigma'} = i[H^{'}_C, \sigma^{'}]$, which can be expressed as a rotation.

$$\dot{\sigma} = \mathbf{\Omega_{eff}(t)} \times \sigma. \tag{5}$$

We consider the effect of a DC error by randomly picking a Rabi frequency from a Gaussian distribution of standard deviation 0.1Ω. The results are shown in the left-hand movie of Supplementary Movie 1. The blue arrow shows the rotation of the Bloch-vector with no Rabi frequency error. The grey vectors show that a spread in Rabi frequencies causes a divergence in the rotation angle of the Rabi oscillation. Consequently, the time ensemble averaged Rabi oscillation is damped, and this is seen as a shrinking of the ensemble averaged Bloch-vector (green).

The Rabi oscillation is less sensitive to errors in the detuning, since the effective Rabi frequency depends quadratically on the detuning for $\Omega \gg \delta$, since $\Omega_{eff} = \sqrt{\Omega^2 + \delta^2}$.

A similar calculation is made for the case of the CCD drive ($\epsilon_m \neq 0$), and is shown as the right-hand movie in Supplementary Movie 1. In the CCD case, the effective magnetic field (Black) performs a rocking motion in the $x^{'}y^{'}$-plane about the $x^{'}$-axis, at a frequency ω_m. This motion limits the spread in the Bloch-vectors (grey), and a clear refocusing behaviour can be seen at a frequency ϵ_m. This locks the Rabi frequency to the modulation frequency ω_m and results in a suppression of the damping of the time-ensemble averaged Bloch vector (green).

Further insight can be obtained by considering the dressed basis, where the Hamiltonian is given by $H^{''}_C$. In this frame, the rotating basis is such that $\sigma^{''} = (0, 0, -1) = |0^{''}\rangle\langle 0^{''}|$ corresponds to a Rabi oscillation with frequency ω_m that starts at time t=0, and $\sigma^{''} = (0, 0, 1) = |1^{''}\rangle\langle 1^{''}|$ is shifted in phase by π, where

$$|0^{''}\rangle = \cos(\frac{\omega_m t}{2})|0^{'}\rangle + \sin(\frac{\omega_m t}{2})|1^{'}\rangle,$$
$$|1^{''}\rangle = -\sin(\frac{\omega_m t}{2})|0^{'}\rangle + \cos(\frac{\omega_m t}{2})|1^{'}\rangle. \tag{6}$$

In the case of an unprotected Rabi oscillation ($\epsilon_m = 0$), for a Rabi-drive that is matched to the clock, ($\Omega = \omega_m$) there is no effective magnetic field, and the Bloch-vector is stationary. However, an error in the Rabi frequency generates an effective field term $\frac{1}{2}(\Omega - \omega_m)\sigma^{'}_x$, causing the Bloch-vector to rotate about $x^{'}$. As before, a spread in the Rabi frequency leads to a divergence in the rotation angle of the Bloch-vector and a damping of the time ensemble average of the Bloch-vector.

When $\theta_m = 0$, the ϵ_m-component gives rise to an effective magnetic field along $z^{''}$. Now, the effect of an error in the Rabi frequency with respect to the clock ω_m is to tilt the effective field away from $z^{''}$. This limits the rotation about $x^{'}$, and limits the admixing of the Rabi oscillation $|0^{''}\rangle$ with $|1^{''}\rangle$. In the lab frame, this results in a small reduction in the contrast of the Rabi oscillation that does not increase with time. The signal will acquire sidebands at the frequencies $\pm\sqrt{\epsilon_m^2 + (\Omega - \omega_m)^2}$.

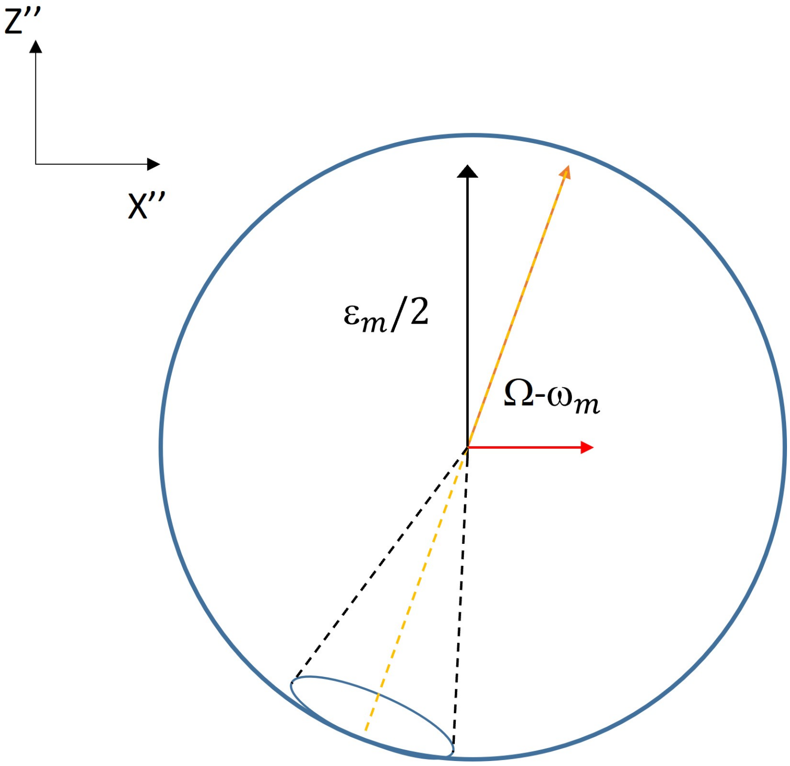

Supplementary Figure 1: In the protected frame, the ϵ_m-term limits the rotation of the Bloch-vector due to an error in the Rabi frequency. A view of the x"z"-plane in the dressed states basis is presented. In the lab frame, the state (0,0,-1) is observed as a Rabi oscillation that is in phase with a clock that starts at time t=0 at a frequency ω_m, see Eq. 17. The state (0,0,1) has a π-phase shift compared to (0,0,-1). In the protected frame, a detuning of the Rabi frequency from the clock frequency ω_m causes a rotation about $x^{''}$. Left unchecked, this will cause a divergence in the phase of the Rabi oscillation, and damping of the Rabi oscillation. Adding an ϵ_m-drive with $\theta_m = \phi = 0$ applies an effective field along the $z^{''}$-direction. Now an error in the Rabi frequency with respect to the modulation frequency, $\Omega - \omega_m$ tilts the effective magnetic field. This results in a precession of the Bloch-vector about the effective magnetic field, limiting the spread in the Bloch-vector.

Supplementary Note 3: Continuous dynamic decoupling of qubit from noise

In this section, we analyze how by using the CCD drive to define a rotating qubit basis, the interactions of the qubit with a fluctuating magnetic field can be shifted in frequency. This can be used to enhance coherence times, by moving to a lower noise frequency regime.

For simplicity, consider a two-level system in a static B-field along z with a Hamiltonian ($\hbar = 1$)

$$H_c = H_0 = \frac{1}{2}\omega_0\sigma_z \tag{7}$$

In addition to the DC B-field, the spin is subject to a fluctuating B-field with a Hamiltonian of the form,

$$\delta H = \frac{1}{2}\sum_{\alpha}\delta\omega_\alpha(t)\sigma_\alpha. \tag{8}$$

To calculate the decoherence rates, we use the Bloch-Redfield equation to calculate changes to the density matrix ρ' in the stationary frame of the control Hamiltonian $H_c(t)$, i.e. $\rho' = e^{iH_ct}\rho e^{-iH_ct}$.

$$\frac{d\rho^{'}}{dt} = -\int_0^t d\tau Tr[\delta H(t), [\delta H(t-\tau), \rho^{'}(t-\tau)]] \tag{9}$$

Making the Born-Markov approximation $\rho'(t-\tau) \to \rho'(t)$, and the upper limit on the integral $t \to \infty$ gives

$$\frac{d\rho^{'}}{dt} = -[\sigma_\alpha(t), [G_\alpha(t), \rho^{'}(t)]] \tag{10}$$

$$G_\alpha(t) = \frac{1}{4}\int_0^\infty d\tau Tr[\delta\omega_\alpha(0)\delta\omega_\alpha^*(\tau)e^{iH_c\tau}\sigma_\alpha(t)e^{-iH_0\tau}] \tag{11}$$

$$F_\alpha(\omega) = \frac{1}{4}\int_0^\infty d\tau Tr[\delta\omega_\alpha(0)\delta\omega_\alpha^*(\tau)e^{i\omega\tau}] \tag{12}$$

$G_\alpha(t)$ describes the correlations in the fluctuating B-field components, which are assumed independent of each other and local time t. The noise power spectrum is described by $F_\alpha(\omega)$. In a basis that is stationary with respect to the control Hamiltonian, H_c, the components of the fluctuating magnetic field are transformed by a rotation at a frequency $\omega = \omega_0$, such that

$$\sigma_\alpha \to e^{i\omega\tau\sigma_\beta/2}\sigma_\alpha^{'}(t)e^{-i\omega\tau\sigma_\beta/2} = \sigma_\alpha^{'}, \alpha = \beta \tag{13}$$

$$= \sigma_\alpha^{'}\cos\omega\tau + \epsilon_{\alpha\beta\gamma}\sigma_\gamma^{'}\sin\omega\tau, \alpha \neq \beta, \tag{14}$$

where $\epsilon_{\alpha\beta\gamma}$ is the Levi-Cevita symbol. This leads to a sampling of the noise spectrum at a frequency ω, which determines the decoherence rates.

For the case of a freely evolving spin, $H_c = H_0 = \frac{1}{2}\omega_0\sigma_z$. The z-fluctuations, leads to phase-flip errors and a dephasing rate proportional to the DC noise.

$$\frac{d\sigma_{x,y}^{'}}{dt}|_z = -4F(0)\sigma_{x,y}^{'}. \tag{15}$$

The x-fluctuations, give rise to bit-flip errors. The rotation with frequency components $\pm\omega_0$ samples the noise spectrum giving rise to contributions from $F(\pm\omega)$. This can be viewed as the energy-cost of a bit-flip.

$$\frac{d\sigma_x^{'}}{dt}|_x = 2i(F_x(\omega_0) - F_x(-\omega_0))\sigma_y^{'}, \tag{16}$$

$$\frac{d\sigma_y^{'}}{dt}|_x = -2(F_x(\omega_0) + F_x(-\omega_0))\sigma_y^{'}, \tag{17}$$

In general, the noise spectrum is stronger at low frequency, leading to a fast damping of the Ramsey interference that is dominated by $\Gamma_2^* = 4F(0)$. Another key point is that the energy-gap induced by the static B_z-field only protects against bit-flip errors. Phase-flip errors are prone to low frequency fluctuations in the size of the energy gap, due to fluctuations in B_z.

Now consider the case of a qubit under a continuous drive. To evaluate the frequency components of the noise spectrum responsible for the damping of the qubit state, we perform a series of rotations on the (x,y,z)-components of the fluctuating magnetic field. Each rotation arises from a term in the control Hamiltonian, and are carried out using the largest energy term first. The results are summarized in Supplementary Table 1. For example, a rotation about the z-axis, leaves the z-component unchanged, whilst mixing the frequencies present in the xy spin-components.

Frame	Rotation	x,y	z
Free	$\frac{1}{2}\omega_0\sigma_z$	(ω_0, ω_0, x)	$(x, x, 0)$
Single drive	$\frac{1}{2}\omega_m\sigma_x^{'}$	$(\omega_0, \omega_0 \pm \omega_m, \omega_0 \pm \omega_m)$	(x, ω_m, ω_m)
Double drive	$\frac{1}{2}\epsilon_m\sigma_z^{''}$	$([\omega_0 \pm \epsilon_m \& \ \omega_0 \pm \omega_m \pm \epsilon_m]; \omega_0 \pm \omega_m)$	$(\omega_m \pm \epsilon_m, \omega_m \pm \epsilon_m, \omega_m)$

Supplementary Table 1: Frequency components of the noise spectrum responsible for damping under free-evolution, single and double drive conditions ($\theta_m = 0$). Under each rotation about the σ_γ axis, with frequency ω_γ the γ spin-component is unchanged. The other spin-components are shifted in frequency by $\pm\omega_\gamma$, and shared between the two spin-components that are not γ. Each entry gives a vector, with the frequency-shifts of each spin component. x indicates that no spin-component is present. & indicates that both frequencies are present. For every frequency presented, the negative value is also present.

In a Ramsey interference experiment, the qubit freely evolves, and the main contributions to damping are $F_z(0)$ and $F_{x,y}(\omega_0)$. Usually, $F_z(0)$ is worse due to low frequency noise.

In a single drive Rabi experiment, the main contributions to damping are $F_x(\omega_0)$, $F_x(\omega_0 \pm \Omega)$, and $F_z(\Omega)$. $F_z(\Omega)$ dominates in the low Ω regime. However, as the Rabi drive increases, the power fluctuations in the drive field lead to an increasing noise at $F_x(\omega_0)$. This can be seen in the experiments, see Fig. 1f in main paper.

In the CCD experiment, the main x-noise terms are $F_x(\omega_0 \pm \epsilon_m)$,$F_x(\omega_0 \pm \omega_m)$, and $F_x(\omega_0 \pm \omega_m \pm \epsilon_m)$ are now shifted away from the drive frequency. The z-noise terms $F_z(\omega_m)$ and $F_z(\omega_m \pm \epsilon_m)$ are shifted away from DC, and provided $|\omega_m - \epsilon_m| \gg$ cut-off frequency of the noise spectrum, the decoherence should be reduced. For further discussion of the protection against noise see Wang et al. [1].

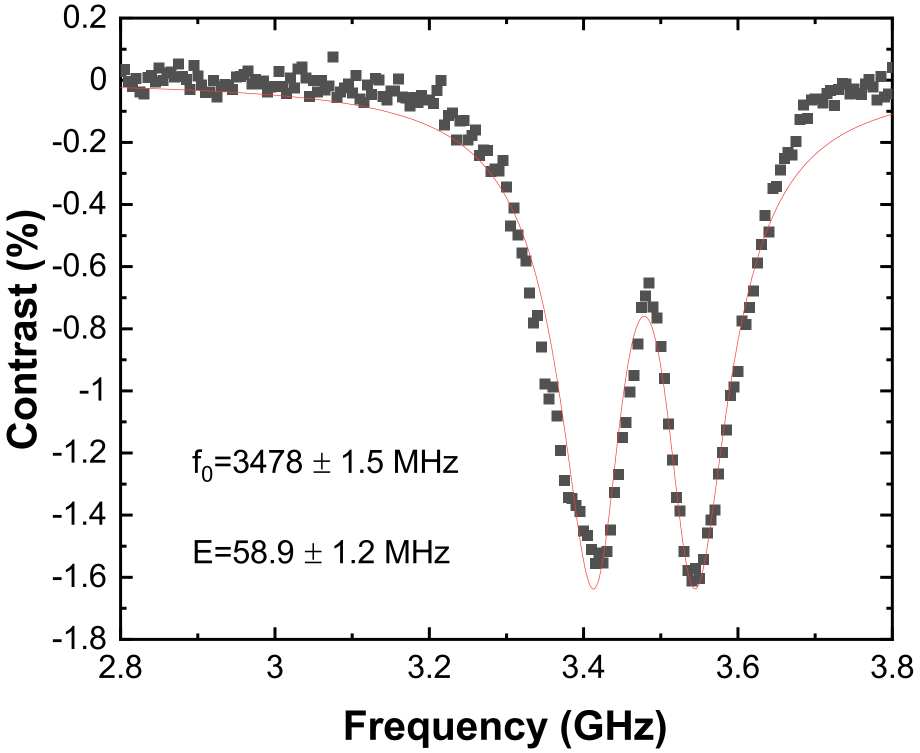

Supplementary Figure 2: Continuous wave optically detected magnetic resonance spectrum with no applied magnetic field. The red line shows a fit to the data, from which a splitting of $E = 59 \pm 1$ MHz is extracted. The fit function used $f(\nu) \propto |\chi_{0+}(f - f_0 - E) + \chi_{0-}(f - f_0 + E)|^2$. This is a sum of the linear susceptibilities of the two ESR transitions from $m_s = 0 \rightarrow \pm$, $\chi(x) = \gamma/(\gamma + ix)$.

Supplementary Note 4: Spin echo measurement at long delay times

In Gottsholl et al. [2], a long-lived component of the spin-echo was reported. Supplementary Figure 3 shows an extension of the spin echo measurement shown in Fig. 1e of the main paper to longer times. We do not observe a long-lived tail. The signal to noise is sufficiently good to have a observed a long-lived tail of similar amplitude to that reported by Gottscholl et al. [2].

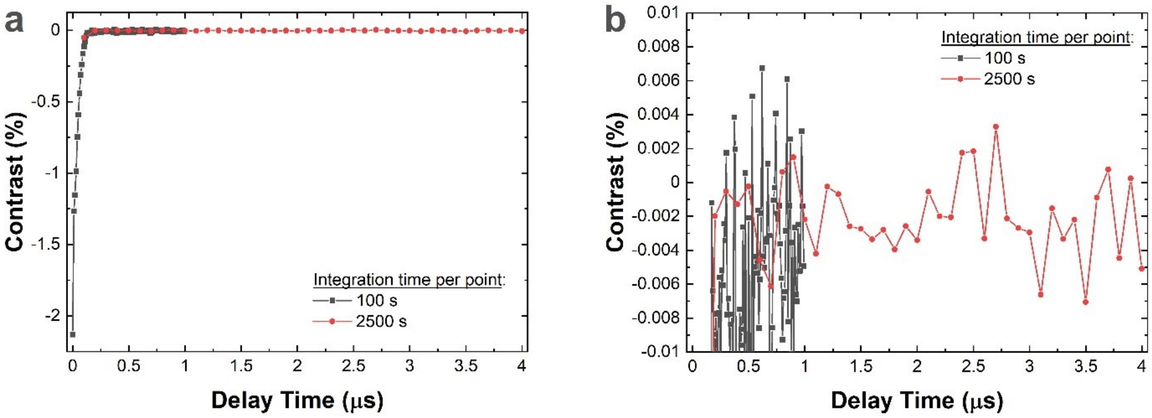

Supplementary Figure 3: **a** Spin echo measurement at long time delay. Beyond 250ns, we do not measure a long-lived tail. **b** A close-up of the data shown in **a**.

Supplementary Note 5: T_1 decay in CCD measurements

In the CCD experiment shown in Fig. 2 of the main text, and similarly in Supplementary Figure 4b, there is a decay of the stabilized Rabi oscillation, but also a decay of the overall contrast. We attribute this to the decay of the longitudinal spin relaxation T_1, which is observed as a consequence of the experimental method. The pulse sequence used in the experiment is shown in Supplementary Figure 4a and consists of three laser pulses, each with a pulsewidth $t_1 = 5$ μs, that are repeated with a period $t_{rep} = 40$ μs. To measure the contrast $C = (I_{sig} - I_{ref})/I_{ref}$, the photoluminescence is sampled during two windows synchronised with two of the laser pulses. The reference intensity I_{ref}, is sampled following a long laser pulse and a short dark period t_0. In this case the spins will be initialised to the bright, $m_s = 0$ state. The signal, I_{MW} is measured following a laser initialisation pulse, and a dark period that is dependent on the MW pulsewidth, τ. In the case of no microwave signal, sweeping τ measures the decay of T_1 as shown by the blue line in Supplementary Figure 4b. This decay is also present during conventional Rabi and CCD measurements, leading to the overall decay of contrast seen in the red and black lines of Supplementary Figure 4b, respectively.

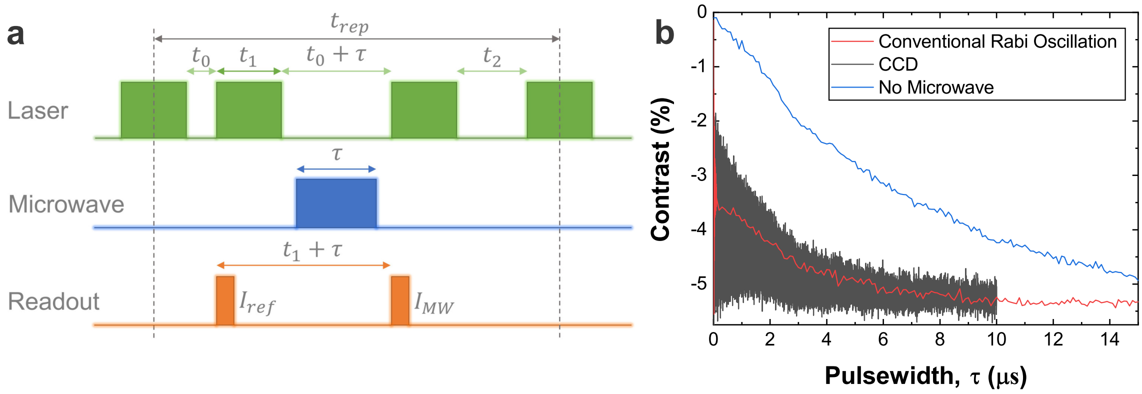

Supplementary Figure 4: T_1 decay during CCD. a Pulse sequence used for Rabi-oscillations and CCD measurements presented in Fig. 2 of the main text. The dark period, $t_2 = t_{rep} - 3t_1 - 2t_0 - \tau$ decreases as the microwave pulsewidth increases in order to maintain a constant overall repetition period. **b** Comparison between Rabi-oscillations and CCD at pulsewidths approaching T_1. With no microwave applied, the contrast decreases due to the transfer of population from the $m_s = 0$ to $m_s = \pm 1$ states.

References

1. Wang, G., Liu, Y. X. & Cappellaro, P. Coherence protection and decay mechanism in qubit ensembles under concatenated continuous driving. *New Journal of Physics* **22,** 123045 (2020).
2. Gottscholl, A. *et al.* Room temperature coherent control of spin defects in hexagonal boron nitride. *Science Advances* **7,** eabf3630 (2021).
