# Supplementary material for: Coherence protection of spin qubits in hexagonal boron nitride
Source: Nat Commun. 2023 Jan 28;14:461. doi: 10.1038/s41467-023-36196-7 (PMC9884286; doi:10.1038/s41467-023-36196-7)
Supplement: Supplementary file 3 — Description of Additional Supplementary Files [file 41467_2023_36196_MOESM3_ESM.pdf]

Description of Additional Supplementary Files

File Name: Supplementary Movie 1: Effect of power fluctuations on time evolution of electron spin under standard Rabi drive (left) and amplitude modulated concatenated continuous dynamic decoupling (CCD) (right). The gray Bloch-vectors are calculated for a random distribution of Rabi frequencies. (left) In the case of a continuous Rabi drive, the distribution in Rabi frequencies results in a divergence of the rotation angle of the electron spin, damping the ensemble averaged Rabi oscillation shown as a green vector. (right) In the case of a CCD drive, the effective magnetic field (black) performs a rocking motion in x'y'-plane about the x'-axis at a frequency ω_m. This motion limits the spread in the Bloch-vectors, and a refocusing behaviour can be seen at a frequency ε_m. This locks the Rabi frequency to the modulation frequencyω_m, suppressing the damping of the ensemble averaged Bloch-vector (green).
