# Supplementary figures and images for: Coherence protection of spin qubits in hexagonal boron nitride
Source: Nat Commun. 2023 Jan 28;14:461. doi: 10.1038/s41467-023-36196-7 (PMC9884286; doi:10.1038/s41467-023-36196-7)

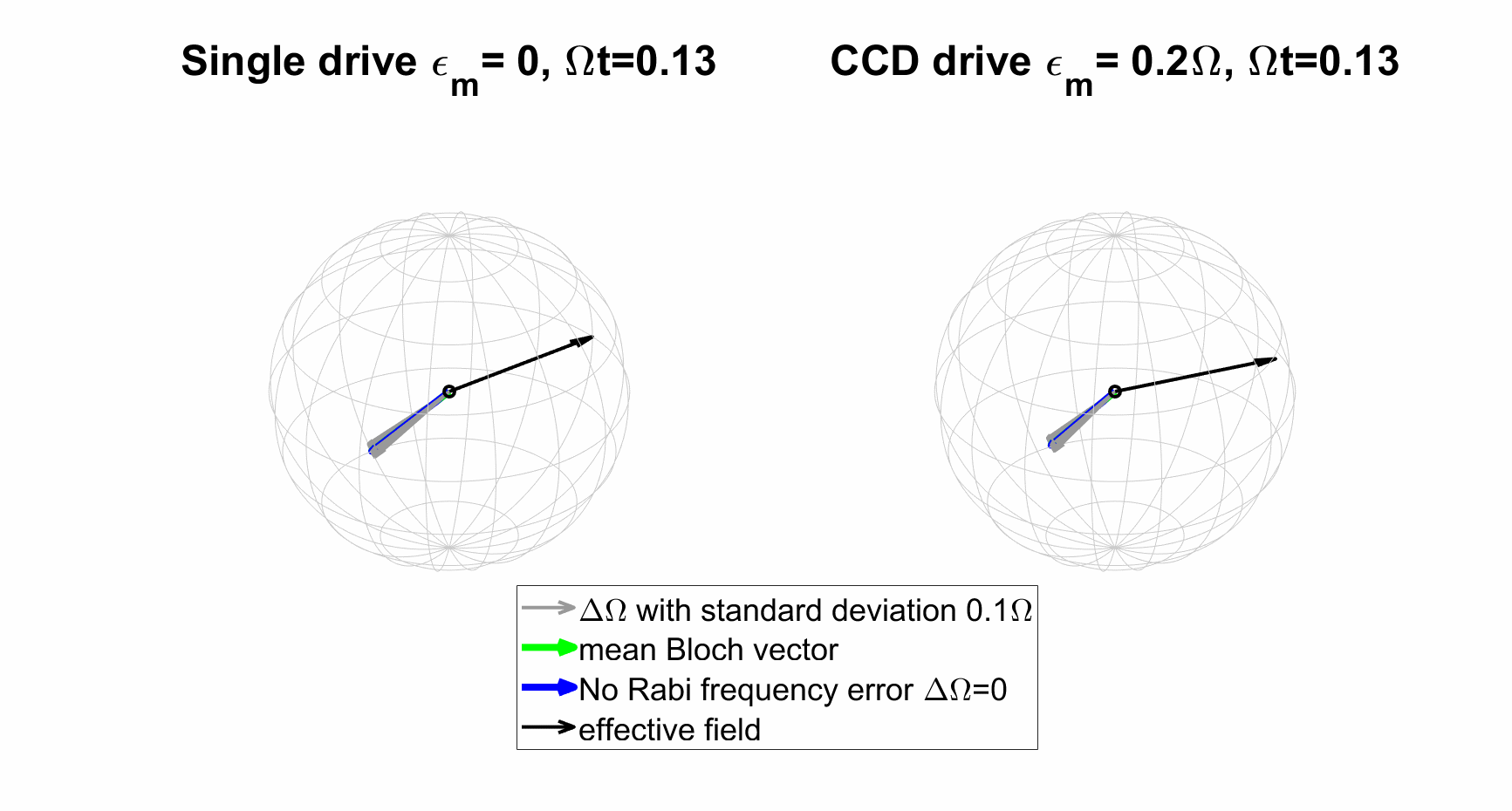

Supplement: Supplementary file 4 — Supplementary Movie 1 [file 41467_2023_36196_MOESM4_ESM.gif]
